# Factors Associated with Male Partner Involvement in Maternity Care in Mbeya, Tanzania

**DOI:** 10.24248/eahrj.v7i2.729

**Published:** 2023-11-30

**Authors:** Getruda Kazimili, Clement N. Mweya

**Affiliations:** a Mbeya College of Health and Allied Sciences, University of Dar es Salaam, Mbeya, Tanzania; b Mbeya Medical Research Centre, National Institute for Medical Research, Mbeya, Tanzania

## Abstract

**Background:**

Male partner involvement in maternity care is critical to improving neonatal and maternal health by reducing maternal mortality, particularly in settings where males play a significant role in decision-making. This study aimed to assess factors associated with male partners involvement in maternity care in Mbeya, Tanzania.

**Methods:**

A community-based cross-sectional study was conducted among men in Mbeya City, Tanzania, from April to June 2021. A semi-structured questionnaire was used to collect information from participants. Male involvement level was measured as low, moderate or high. X^2^ test and multinomial logistic regression models were applied to determine association between male involvement levels and related factors.

**Results:**

A total of 201 males participated in the study. The overall level of male involvement during antenatal care, labour and delivery and postnatal care indicated that 44 (21.7%) had a high level of involvement, 116 (58%) had a moderate level of involvement and 41 (20.3%) had a low level of involvement. Demographic and health facilities factors indicated a significant association with male partner level of participation (*P<.001*). The likelihood of a man accompanying the partner was significantly associated with staff attitude and the time spent at the health facility (AOR 1.726 at 95% CI 1,394-2.136 *P<.001*).

**Conclusions:**

Findings indicated a generally moderate level of male partner involvement as a critical concern that can accelerate the decline in maternal mortality and improve maternal health. Enhancing the male-friendliness of health facilities in terms of infrastructure, organisation of services and staff attitudes, as well as educating the community, particularly men, to sensitise them to the negative attitudes toward male participation in maternity care, can increase male participation.

## BACKGROUND

Men are essential stakeholders and should be considered in maternal and child health.^1^ Even though men have important decision-making roles related to maternal and child health issues, maternal and child health is viewed as a woman's affair in many sub-Saharan African countries, including Tanzania.^2–4^ The positive impact of male involvement in maternal and newborn health is not unique to low-resource settings. In high-resource settings such as the United States of America, Israel, Great Britain, and Sweden, male involvement has positively affected children's cognitive development.^5–7^

In recent years in Africa, increasing efforts have been made to involve men in maternity care, including family planning, eliminating Human Immunodeficiency Virus (HIV) Transmission from the mother to the child, and safe motherhood.^8–10^ The primary aim of these programs was to reduce maternal and child mortality.^11,12^ Studies have shown that male involvement in maternity care is associated with improved maternal and child health outcomes.^7,13,14^ However, the proportion of male participation in maternity care in Sub-Saharan Africa remains low.^15–17^ Male partners in African communities are typically the decision-makers in all matters about families.^18,19^

In low-resource settings such as Tanzania, Nepal and Malawi, men who want to be positively involved in maternal and newborn health can face substantial barriers to local expectations of male roles.^1,5^ For example, a male reported experiencing social stigma against male involvement. The male expressed feelings of being ignored by facility staff. Men also described encountering actual or perceived policies restricting their movement and presence where women's health services are delivered in Nepal, Kenya, Rwanda, and Ghana.^5^ Despite evidence and calls for action, implementers have struggled to develop programs to increase male involvement due primarily to perceptions and implications of gender norms. Implementation challenges are also linked to a lack of research directly involving participation of men in prenatal, labour, delivery, and postpartum care.

The maternal mortality rate in developing countries continues to be high (240/100,000 live birth), while that of developed countries is lower (16/100,000 live births).^20^ Sub-Saharan Africa suffers from the highest MMR of 533 deaths per 100,000 live births. Tanzania, a Sub-Saharan African country, has a MMR of 432 per 100,000 live births, a long shot from its target of 193 per 100,000 live births.^21^ Some progress has been made since 2000 when this goal was set, but the rate of decline has been prolonged. Despite different programs and interventions, this poor performance has persisted in reducing maternal mortality. Mbeya City is not exempt from this national problem. The MMR in Mbeya region is around 776 per 100,000 live births.^22^ One of the key factors contributing to the high mortality rate is women's low utilisation of health services.

In our socio-cultural setting, men wield a lot of power in decision-making in the household, and they play a vital role in women's health-seeking behaviour. Funding and permission to seek maternity care often come from a male partner. Male partner involvement in maternity care is low and has contributed to the slow decline in maternal mortalities. Various factors determine male partner involvement in maternity care. These include socio-demographic, cultural, economic, religious program, and health facility factors.^1,5,7^ In Tanzania, little work has been done to understand male involvement in maternity care. The levels of male partner involvement in maternity care and aspects that determine this in the study area, Mbeya City Council, have not been elucidated. Most researchers focus on women, but research must also be targeted to get their perspective if men are involved.

## METHODS

### Study Area

**FIGURE 1:**
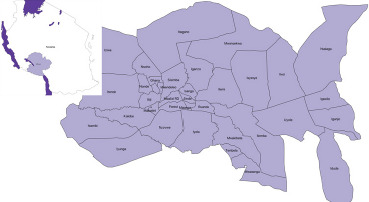
Map of Mbeya City

This study was conducted in Mbeya City, southwest highlands of Tanzania. Mbeya City is administratively divided into 36 wards. The area of Mbeya City is bordered to the north by Mbeya Rural District, to the east by Rungwe District, to the south by Ileje District and to the west by Mbozi District (Figure 1). Mbeya City has a total of 50 health facilities, of which five (5) are hospitals, eight (8) health centres and thirty-seven (37) dispensaries.^23^ Because our study was conducted in 2021, before the current National Census, we used population data based on the 2012 National Census. Mbeya City had 364,934 inhabitants, of which 182,620 (47%) were males and 202,659 (53%) were females.^22^

### Study Design

A community-based cross-sectional study was conducted in Mbeya City. The study was carried out from April to June 2021. The multistage sampling method was used to select wards from Mbeya City using a table of random numbers from which eligible men were included in the study.

### Study Population

The study involved men aged 18 years and above with children of 5 years and below. Those men who did not have children or whose last child was above five years were excluded from the study.

### Data Collection

Data were collected using a structured questionnaire in Swahili language for easy understanding. The participants were given questionnaires and filled out that questionnaire under minimal supervision to ensure freedom and confidentiality after being sure they could fill it out.

### Sample Size Estimation

The minimum sample size (N) required for this study was 201, calculated using a Cochran formula for cross-sectional studies.^24^ The prevalence (P) of 16% was recorded from the Tanzania Demographic and Health Survey of 1996.^25^

N = Z^2^ P (1 – P)/E^2^ (N = Minimum sample size required, Z = Normal standard deviation, at 95% confidence interval = 1.96, P = Prevalence rate, E = Standard maximum error)

### Statistical Analysis

Data were entered and analysed using IBM SPSS Statistics 23.0 (IBM Corp., Armonk, NY, USA). The descriptive statistics for categorical variables were expressed in numbers and percentages. The X^2^ test was used to test the association between demographic factors and levels of male partner involvement. Multinomial logistic regression was used to test the likelihood of a man accompanying the partner more than once in assessing staff attitude and the time spent at the health facility during the first visit. A *P* value of less than .05 was considered statistically significant.

### Ethical Approval and Consent to Participate

Ethical approval was obtained from the University of Dar es Salaam, Mbeya College of Health and Allied Science Research Ethical Clearance Sub-Committee (Ref No. AB 458/482/02/476. Permission to conduct the study was obtained from the Regional Administration, District Administrative Secretary (DAS) of Mbeya and Ward Executive Officers (WEOs) of respective wards. Also, the Chairpersons of the streets were notified. Informed consent was sought and obtained from all participants before enrollment in the study. Confidentiality and privacy of participant's information were observed during the interview. Respondents were interviewed separately in a confidential manner, and names and other identifying information were not recorded in the questionnaires.

## RESULTS

### Socio-demographic Characteristics of Respondents

Two hundred and one (201) men participated in the study, with a response rate of 100%. The average mean age of study participants was 30.4 years, with a standard deviation (SD) of 8.2. Most participants, 174(86.6%) were married. The majority of respondents, 104(51.7%) were between 26 and 33 years old. One hundred and twenty, (59.7%) respondents were self-employed. The majority of respondents, 128(63.7%) had a secondary level of education and 12(6%) never attended formal education. Respondents with more than four children were 89(44.3%) and most respondents, 157(78.1%) were Christian. The socio-demographic characteristics of the study participants are shown in Table 1.

**TABLE 1:** Distribution of Sociodemographic Characteristics of Study Participants (N=201)

Sociodemographic characteristics	Frequency	Percentages (%)	P Value
Age of respondents			
18–25	30	14.9	<.001
26–33	104	51.7	
34–41	47	23.4	
>41	20	10	
Marital status			
Married	174	86.6	.02
Cohabitating	11	5.5	
Divorced	12	6	
Single	4	2	
Level of education			
No formal education	12	6	.04
Primary school level	45	22.4	
Secondary school level	128	63.7	
Collage and a higher level of education	16	8	
Main occupation			
Unemployed	17	8.5	<.001
Self-employed	120	59.7	
Employed	64	31.8	
Number of children			
1	28	13.9	
2–4	84	41.8	<.001
>4	89	44.3	
Religion			
Christian	157	78.1	
Muslims	44	21.9	

### Male Involvement in the Antenatal Care of their Partners

**FIGURE 2:**
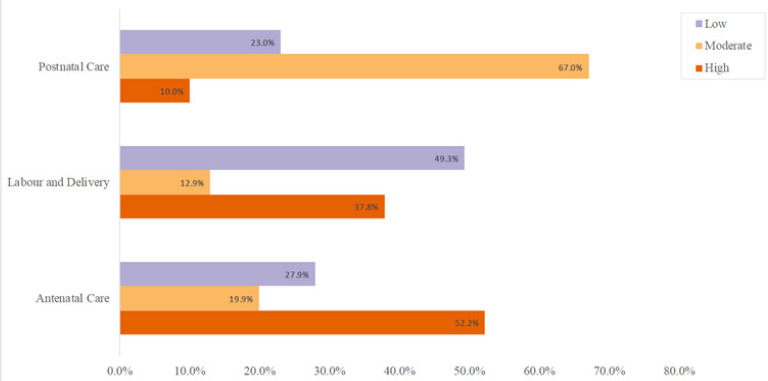
Male Involvement in their Partner's Antenatal Care

The most recent pregnancy was planned among 153 (76.1%) cases and unplanned in 48(23.9%). One hundren eighty-six (92.5%) men knew their partners attended the antenatal clinic and understood the specific facility they had the care. However, 15(7.5%) were not involved in the decision-making on where the women received antenatal care. Fifty-four (26.9%) participants accompanied their partners at least once to the antenatal clinic and 147 (73.1%) never did. Of those who accompanied their partners, 39 (72.2%) did so once, 11(20.4%) made two to three visits, and only 4 (7.4%) did so four or more times. Male involvement level during antenatal care indicated that 105 (52.2%) participants had a high level of involvement, 40 (19.9%) had a moderate level of male involvement and 56 (27.9%) had a low level of male involvement (Figure 2).

### Male Partner Involvement during Labour and Delivery

The number of participants who lived with their partners at the time of labour and delivery was 147(73%), a slightly lower number than during pregnancy. However, a high percentage (40%) accompanied their partners to their health facility during labour and delivery. Of those who did not accompany their partners, 68(56.2%) delegated someone else to accompany the woman to the health facility. Of those who accompanied their partners to health facilities, only one participant (1.3%) indicated that male partners were allowed in the labour and delivery rooms. When asked about their opinion on who should be allowed into the labour and delivery room if only one person could be allowed, 76(38%) felt the husband should be the one, 99(49.3%) said it should be the woman's mother, 40 (20%) said it should be man's mother and 4 (1.8%) said a sibling should be allowed in. A joint plan for labour and delivery was made by 161(80%) participants, but 40(20%) made no prior plans. Male involvement level during labour and delivery indicated that 76(37.8%) participants had a high level of involvement, 26(12.9%) had a moderate level of male involvement and 99(49.3%) had a low level of male involvement (Figure 2).

### Male Involvement in Postnatal Care

Eighty-three (41.3%) participants did not live with their partners after delivery, whereas 118(58.7) did. Of the women who did not live with their partners, 170(85%) lived with their mothers, 20(10%) lived with their mother-in-law and 11(5.5%) lived with their sibling. One hundred eighty-five (92%) never accompanied their partners to the postnatal clinic, but 16(8%) did. More than a quarter of participants, 56(28%) had discussions on issues relating to the postnatal period, like family planning with their partners but 145(72%) did not. Only 18 (9%) had any such discussions with their partner's health provider, but 183 (91%) did not. (69%) made prior plans for their partner's postnatal care, and (31%) were not. All participants provided some form of physical or financial support to their partners. Male involvement level during postnatal care indicated that 20(10%) participants had a high level of involvement, 135(67%) had a moderate level of male involvement, and 46(23%) had a low level of male involvement (Figure 2). The level of participation in all three aspects of maternity care together indicated that participants who had high, moderate and low levels of involvement were 44(21.7%), 116(58%) and 41(20.3%), respectively.

### Demographic Factors Influence on Male Partner Involvement in Maternity Care

Participants in the 18–25 years group had the highest proportion (55%) of male involvement, whereas the lowest level of male involvement was among participants in the 26–35 years group (X^2^=47, *p<.001*). Those who were married had the highest level of male involvement (36%). In comparison, single participants had the highest proportion (78%) of men with low participation, followed by those who were cohabitating (48%) and those who were divorced (31.4%). Those with no formal education had the highest proportion of male involvement (62.1%) and those who had completed college and above had the lowest proportion of low male involvement (4%). Those unemployed had the highest proportion (60%) of male involvement. Public servants had a slightly higher proportion of male involvement (28.6%) than those in the private sector (23%). Participants with 2 to 4 children had the highest proportion (36%) of a high level of involvement. Those with their first and five or more children had 10% and 13% of high-level involvement, respectively (X^2^= 94, *P<.001*).

### Health Facility Factors Influence on Male Partner Involvement in Maternity Care

The likelihood of a man accompanying the partner more than once was significantly associated with his assessment of staff attitude and the time spent at the health facility per visit (AOR 1.726 at 95% CI 1,394-2.136 *P<.001*). Of those who found the staff unfriendly, 97.9% accompanied their partners once, and 2.15% accompanied them twice or more. More than half (60%) of those who found the staff friendly accompanied their partners two or more times. Of those who assessed the time spent at the health facility to be too long, 95.2% accompanied their partners once, and 4.3% did so twice or more. Seventy-nine per cent (79%) of those who accompanied their partners more than once found the time spent at the health facility reasonable.

## DISCUSSION

Maternity care (antenatal, intrapartum and postnatal) has often been seen as a women's issue. Men, women and even health workers usually see that pregnancy and delivery are a woman's domain, so the emphasis has been on women, with low male involvement.^8,26^ However, men have been identified as key to reducing maternal mortality and improving maternal health in developing countries.^27,28^ Communication among couples about family planning positively impacted women's contraceptive behaviour.^29^ Findings from other studies indicate that male involvement in maternity care varies across communities and countries, with various factors determining the level of involvement in studies.^14,30,31^ These factors include socio-demographic, cultural or even inherent health delivery systems. Awareness of these factors is essential in formulating policies and providing services that encourage male involvement and remove barriers to their participation in maternity care.

The overall findings from this study show that most men from Mbeya are moderately involved in the maternity care of their partners. Twenty-one per cent were highly involved. The high-level participation proportion was similar to findings in Mbale District, Uganda, which found that 26.0 % of the participants had a high level of involvement. ^14^ However, it was found that 74.0% of the participants had a low male involvement index,^14^ categorised their male involvement index into high and low with no moderate group. Another study in the same country found that male involvement in antenatal care was high (67.2%). About 71.9% accompanied their partners to antenatal clinics at one point during the pregnancy period. Of this, 45.7% did so four or more times, 35.7% went 2 to 3 times and 18.6% went only once. Factors such as staff attitude, time spent at the clinics, age, educational level, monthly income level, living with a partner during pregnancy, distance to the clinic, and community acceptability were all statistically significant to male involvement.

This study found that men (26.9%) reported making joint decisions regarding antenatal care (ANC) with their partners. This finding differs significantly from previous studies where couples' collective decision-making was reported as low as 9% in Nigeria and 28.6% in India.^32,33^ This variation could be due to cultural differences and limited exposure to safe motherhood initiative programs. Male involvement in making decisions refers to men having knowledge and participating in maternal health issues. That is, acting with women as partners and supporting decisions and activities to improve women's health. It encourages communication among couples.^14,27^ It does not imply male dominance and seeks to decrease female autonomy. Male unilateral decision-making may reduce women's healthcare utilisation.^34^ There has been fear that male involvement may lead to patriarchal domination and decrease female autonomy.

The highest male involvement was in ANC and during labour and delivery. This is probably because this period is seen as a crucial time and rightly so because most maternal mortalities occur around this period. The postnatal period had the lowest level of male involvement. When a woman has a safe delivery of a healthy baby, it is often considered the end of the pregnancy. So, less attention is given to the postnatal period. Although the proportion of men with low involvement was low, the key points that involved contact with the health facility and health professionals were the points that scored the lowest. A study in central Tanzania found that men's involvement in ANC was high (53.9%). The majority, 89%, of respondents made joint decisions to seek ANC. More than half (63.4%) of respondents accompanied their partners to the antenatal clinic at least once. Less than a quarter (23.5%) of men were able to discuss issues related to pregnancy with their partner's healthcare providers. About 77.3% of respondents provided physical support to their partners during the antenatal period. Factors influencing men's involvement in ANC were occupation, ethnicity, religion, waiting time, information regarding men's participation in ANC and men's perception of the attitude of health care providers.

Men accompany the partner to the health facility and discuss the female partner's maternity care with health care providers. This is a cause for concern because, during contact and discussions with health professionals, men can acquire the needed knowledge, behavioural and attitudinal change that can positively impact their contribution to reducing maternal morbidity and mortality. This was lower than findings made in the study in Kabale, Uganda, which found that 42.9% of the women had been accompanied by their husbands to the antenatal clinic 43.4% to the labour ward.^8^ However, the finding was higher than in Mbale, Uganda,^14^ where only 5% of the men accompanied their spouses to the antenatal clinic. More than a quarter (29, 9%) of the men reported accompanying their partners at least once to an ANC visit. This is lowest than previous studies in Uganda and Nigeria, where the proportion of male involvement in ANC was 65.4 and 63%, respectively.^35,36^ Shared cultural values on gender roles among African societies could explain the observed similarities in these findings.^26,37^

In this study, of those who accompanied their partners to ANC majority, 72.2% were accompanied once. Continued health education given to women and men at reproductive and child health clinics, coupled with the influence of safe motherhood initiatives, including ongoing media campaigns such as the “Wazazi Nipendeni” project, may have further contributed to high male involvement increased couples' joint decision-making on ANC issues. This could explain the high proportion (77.3%) of men who reported offering support to their partners, including relieving them from some household chores during pregnancy. Previous studies have reported a low proportion of men supporting their partners.^8,30^

In this study, more than two-thirds of respondents (77.8%) who accompanied their partners to an ANC visit reported spending too long in healthcare facilities waiting for services. This is likely to discourage men from coming to ANC in subsequent visits. Studies in other parts of Africa show that the longer the time spent waiting for services, the fewer the chances for men to be involved in ANC services.^38–40^ Thus, healthcare providers and program implementers should take appropriate action to advocate and encourage men's involvement in ANC. During ANC, pregnant women and their partners are given health education. This may result in a more significant outcome on maternal health behaviours than when women receive this education alone.^27^ It is understood that education and health services provided during antenatal period can reduce pregnancy and delivery complications and improve birth outcomes.^41^ Thus, if men and women miss this opportunity during ANC, it is unsurprising that SDG 3 is not achieved. Previous studies have reported a low proportion of men offering such support to their partners.^8,30^ It has been observed that involving partners in maternal health care and encouraging joint decision-making among couples may be essential in improving men's involvement and couples empowerment.^42^

## CONCLUSION AND RECOMMENDATIONS

Findings from our study indicated a generally moderate level of male involvement in maternal care. A high level of male partner involvement was recorded during ANC, during labour and delivery as compared to postnatal care. Demographic characteristics, the health facility and health care providers factors have been shown to influence male partner involvement in maternity care. There is a need for a concerted effort for all these stakeholders to improve male involvement in maternity care. The healthcare delivery system is best placed to spearhead this effort by providing facilities with infrastructure designed to accommodate and welcome men to participate in maternity care. Attitudinal change among healthcare providers to a friendlier attitude will foster greater male involvement. The organisation of service delivery in a sensitive way to the time constraints of the male partners will encourage more men to accompany their partners to the health facilities. Findings from our study recommend that these critical issues brought to the fore will help formulate policies that remove barriers to male participation in maternity care and translate into greater utilisation of health services by women. Ultimately, this should result in improved maternal health and reduced maternal mortality and accelerate the achievement of SDG - 3
